# Descriptive forest: experiments on a novel tree-structure-generalization method for describing cardiovascular diseases

**DOI:** 10.1186/s12911-023-02228-x

**Published:** 2023-07-28

**Authors:** Peera Liewlom

**Affiliations:** Department of Computer and Information Science, Faculty of Science and Engineering, Kasetsart University, Chalermphrakiat Sakonnakhon Province Campus, Sakonnakhon, 47000 Thailand

**Keywords:** Information Science, Medical Informatics, Data Mining, Cardiovascular Diseases

## Abstract

**Background:**

A decision tree is a crucial tool for describing the factors related to cardiovascular disease (CVD) risk and for predicting and explaining it for patients. Notably, the decision tree must be simplified because patients may have different primary topics or factors related to the CVD risk. Many decision trees can describe the data collected from multiple environmental heart disease risk datasets or a forest, where each tree describes the CVD risk for each primary topic.

**Methods:**

We demonstrate the presence of trees, or a forest, using an integrated CVD dataset obtained from multiple datasets. Moreover, we apply a novel method to an association-rule tree to discover each primary topic hidden within a dataset. To generalize the tree structure for descriptive tasks, each primary topic is a boundary node acting as a root node of a C4.5 tree with the least prodigality for the tree structure (PTS). All trees are assigned to a descriptive forest describing the CVD risks in a dataset. A descriptive forest is used to describe each CVD patient’s primary risk topics and related factors. We describe eight primary topics in a descriptive forest acquired from 918 records of a heart failure–prediction dataset with 11 features obtained from five datasets. We apply the proposed method to 253,680 records with 22 features from imbalanced classes of a heart disease health–indicators dataset.

**Results:**

The usability of the descriptive forest is demonstrated by a comparative study (on qualitative and quantitative tasks of the CVD-risk explanation) with a C4.5 tree generated from the same dataset but with the least PTS. The qualitative descriptive task confirms that compared to a single C4.5 tree, the descriptive forest is more flexible and can better describe the CVD risk, whereas the quantitative descriptive task confirms that it achieved higher coverage (recall) and correctness (accuracy and precision) and provided more detailed explanations. Additionally, for these tasks, the descriptive forest still outperforms the C4.5 tree. To reduce the problem of imbalanced classes, the ratio of classes in each subdataset generating each tree is investigated.

**Conclusion:**

The results provide confidence for using the descriptive forest.

## Background

Cardiovascular diseases (CVDs) are a group of disorders of the heart and blood vessels and are the primary cause of worldwide human deaths [1]. In 2019, CVDs accounted for approximately 17.9 million deaths (32% of global deaths). CVDs include coronary heart disease, cerebrovascular disease, peripheral arterial disease, rheumatic heart disease, congenital heart disease, and deep vein thrombosis and pulmonary embolism.

Available patient datasets are regularly updated to obtain additional knowledge about CVDs and understand the risk factors related to them [2]. Knowledge on CVDs is commonly acquired through decision or classification trees that describe CVD-related features [3–5].

C4.5 [6] is an algorithm that generates decision trees [7]. A C4.5 tree comprises a root node, decision nodes, leaf nodes, and edges (or branchs). Moreover, each leaf node is a class and the other nodes are features. Each edge branch from a node is node’s feature value. All edges start from the root node, sequentially move to other nodes, and finally reach a leaf node. The route from the root node to the leaf node represents the related features of the class in that leaf node. Thus, a decision tree can describe the features related to a class and is applicable to the descriptive tasks of CVDs [8]. Many studies have used various tree algorithms, such as ID3 [9], M5P [10], and C4.5 [6], to describe hidden knowledge in datasets.

However, the accuracy of tree predictions depends on the subpopulation of the training data [11]. Some studies have proposed using available CVD datasets to ensure accurate predictions; however, this method does not yield optimally accurate predictions because the available data are extremely limited compared with all CVD patients. Stiglic et al. [12] used trees to describe hidden knowledge by focusing on scientific tasks, the ability to explain related features, and class value. However, their study required a clear tree structure that is not extremely complex to describe the perspicuous knowledge.

A tree structure is suitable for describing the primary topic of the root node or the primary and respective minor factors of the class of interest. This description is based on a greedy algorithm [13] for creating trees [14]. The root node is the feature best related to the classes. Subsequently, the other nodes in each route are the ordered features best related to the classes. All descriptions using a tree have a bias from the root node, or only a main topic.

However, the main topics or primary factors in CVDs are complex. The causes of the disease are determined by many simple or complex factors. For CVDs, the risk for each patient is determined through various environmental factors, and “the greater risk for CVDs is attributed to disparity in risk factors” [3]. CVD datasets have various related factors from different environments. Thus, we must use many trees as primary and related factors to describe the risk for each patient. If we use only the optimal tree to describe the risk, all risk explanations will be biased by one primary topic or primary factor at the root node. Moreover, other primary and related factors will be described in a highly complex tree structure that is difficult to understand or useless for descriptive scientific tasks.

Some studies have used trees to discover the main topics or primary features of a dataset. Son et al. [8] reported the primary features by identifying frequent features from trees constructed from training datasets using 10-fold cross-validation. This study employs an efficient method, using some trees to describe the primary features and all the trees for prediction tasks only. Scheurwegs et al. [15] reported the primary features by selecting pivot points from the internal scoring metric in random forests. This is because a method that uses all the trees for descriptive tasks is difficult to clearly define.

The random forest algorithm [16] uses a policy similar to the research problem. For prediction tasks, many trees are used to avoid overfitting in the heart disease dataset [15]. Compared to using one tree, using a random forest for prediction task yields superior accuracy from heart disease datasets [17–19]. These results confirm the benefits and usability of random forests for prediction tasks; however, these trees are based on random features and training datasets. In addition to accuracy in prediction tasks, we need the ability of trees for descriptive tasks. Nevertheless, trees generated from random forests are difficult to use in descriptive tasks.

A hybrid technique using random forests involves employing various feature-selection techniques to discover the related features of a dataset. For example, Mohan et al. [20] discovered the related features using an a priori algorithm. Ghosh et al. [21] discovered the related features using the Relief and LASSO feature-selection techniques. Ashri et al. [22] discovered the related features using a genetic algorithm. All feature-selection techniques using random forests are used for classification tasks and have difficulty explaining the features.

Moreno-Sanchez [23] used ensemble trees to describe the primary features by voting and selecting features from ensemble trees (or feature-selection tasks). Subsequently, these features were used to generate a new decision tree to describe the knowledge hidden in a dataset, one compact tree for a descriptive task. However, we must first focus on discovering the main topics or primary features from a dataset; afterward, each main topic can be used to generate a tree. All trees are used for new ideas for descriptive tasks.

These backgrounds indicate that complex knowledge is hidden in integrated datasets. One dataset contains many primary features, each related to other features. A clear example of this problem is the CVD dataset. Each patient has different primary and related features. When performing a descriptive task using decision trees, a single tree is insufficient for the features related to the CVD risk of all patients.

To address this paradigm, we propose a novel method that helps find primary features or main topics in a dataset. Each primary feature is the root node of a tree to which all node members are related. For a descriptive task, the size of each tree is found using the proposed policy of least prodigality for the tree structure (PTS). All trees are combined to explain or describe the CVD risk of each patient in the dataset and to gage the overall CVD risk of the dataset. When used together, the trees form a descriptive forest.

As defined in this study, the descriptive forest is characterized by the 1) tree-structure generalization, 2) use of many trees, and 3) discovery of the primary features (which differs from feature section). Table 1 compares these characteristics of the descriptive forest with those of previous approaches sharing a few similar characteristics.

**Table 1:** Characteristics of a descriptive forest and related works

**Related work**	**For prediction**	**For description**	**Generalization of tree structure**	**Number of trees**	**Primary-feature discovery**	**Feature selection**
SON ET AL. [7]	yes	yes	tree from feature selection	1		rough set attribute reduced on 10-fold cross-validation
STIGLIC ET AL. [12]	yes	yes	tuning the tree fitting in one screen	1		
SCHEURWEGS ET AL. [15]		yes		many	selecting primary features using the internal scoring metric in Random Forest	
BREIMAN [16]	yes			many		
JOLOUDARI ET AL. [18]	yes	yes	rules selecting from parts of trees	many		ranking of predictor significant
MOHAN ET AL. [20]	yes			many		apriori algorithm
GHOSH ET AL. [21]	yes			many		Relief and LASSO
ASHRI ET AL. [22]	yes			many		genetic algorithm
MORENO-SANCHEZ [23]	yes	yes	decision tree constructed from feature selection at the maximum level 3	many for prediction, 1 for description		feature-important measure
A DESCRIPTIVE FOREST		yes	the least PTS	many	association-rule tree with a constraining rule	

The previous works outlined in Table 1 used trees for predictive and descriptive tasks. In these works, the ability to perform descriptive tasks was improved by generalizing the tree structure or discovering the primary features.The descriptive forest alone proposes the use of multiple trees along with a generalized structure for performing the descriptive tasks.

In this study, we show the existence of many trees in a large dataset integrated from various minor datasets. First, we show that each dataset has a tree that fits it. Second, we demonstrate the restructuring of trees when consolidating one tree generated from an integrated dataset. However, a single tree is complex and difficult to explain or understand, thus presenting a research problem to be solved in this study: discovering related trees from an integrated dataset.

In a previous study [24], we applied an association-rule tree [25] to discover the main topics or primary features from a CVD dataset. These main topics were integrated into a fishbone diagram using multiple data mining techniques. The association-rule tree started from a constraining rule ∅ ⇒ {Heart Disease = Yes}, where ∅ is the null itemset with questions such as “What has itemsets related to heart disease?” Only rules that had (1) itemsets with a replacement at ∅ and (2) a strong relationship were considered nodes in the orderly tree. A strong relationship was determined by a slope of interestingness [25], developed from the principle of the “profitability-of-interestingness measure” [26].

In this study, we used an association-rule tree [25] to discover the main hidden topics or primary features from a CVD dataset. The association-rule tree uses the slope of interestingness to avoid tasks to identify suitable values for the minimum support and confidence. Subsequently, each main topic is considered the boundary node acting as the root node of the C4.5 tree (generated from related instances in the dataset) with the least PTS to generalization of the tree structure for descriptive tasks. All trees work together to build a new idea for descriptive tasks, which is known as the descriptive forest.

The results of the descriptive forest on a descriptive task are qualitatively and quantitatively compared with those of a C4.5 tree.

The qualitative comparative study shows how the tree structures differ between the descriptive forest and a single C4.5 tree with the same policy of tree-structure generalization (least PTS). It also compares the details of the explanation between the descriptive forest and a single C4.5 tree on the same selected objects in the same database.

The quantitative comparative study shows the coverage and correctness of the explanation found by the descriptive forest and single C4.5 tree on the whole dataset. As the prediction measures, we apply the recall as a proxy of coverage and the accuracy and precision as proxies of correctness. Note that these measures determine the usability of explanations on the whole dataset and not the efficiency of predicting classes of new objects.

The descriptive forest was evaluated on a compact dataset containing 918 records of a heart failure–prediction dataset with 11 features collected from five datasets [27]. The acceptability is then checked on a larger dataset containing 253,680 records of imbalanced classes of a heart disease health–indicators dataset [28], adapted from the Behavioral Risk Factor Surveillance System 2015 (BRFSS 2015) [29]. This dataset has 22 features and only 23,893 records in the class HeartDiseaseorAttack = Yes (HDA = Yes), which is less than 10% of the whole dataset. Moreover, this dataset contains 276 times more records than the pilot CVD dataset, a heart failure–prediction dataset.

This study aims to include many decision trees from an integrated CVD dataset for descriptive tasks. The remainder of this paper is structured as follows. Next section presents the methods for proving the existence of the research problem, construction of a descriptive forest, and comparative study that proves the usability of the descriptive forest. The sections of results, discussion, and conclusions are presented, respectively.

## Methods

Our research methodology comprises five phases: Phase I involves analyzing the existence of many tree structures generated from an integrated CVD dataset, and Phase II involves constructing the descriptive forest. In Phase III, each tree structure in the descriptive forest is compared with a single C4.5 tree. Phase IV involves comparing the usability of the descriptive forest and a single C4.5 tree, and Phase V involves applying the proposed method to a larger dataset.

### Phase I: analyzing the existence of many tree structures generated from an integrated CVD dataset

The dataset suitable for resolving this study’s research problem must be collected from various datasets. This is because most datasets can generate trees for different analyses. Therefore, we selected the CVD dataset [27] from Kaggle.com, which was collected from five datasets from the UCI Machine Learning Repository [30]. The dataset had 1,190 instances, with 11 features and one class feature ({Heart Disease = Yes} and {Heart Disease = No}). However, it had only 918 instances after filtering for duplicates [27].

The nonduplicated CVD dataset was compared with the UCI datasets, and four datasets were deemed sufficient to study the hidden trees in this CVD dataset. From them, we identified 302 nonduplicated instances in the 303 Cleveland dataset, 293 nonduplicated instances in the 294 Hungarian dataset, 123 nonduplicated instances in the 123 Switzerland dataset, and 199 nonduplicated instances in the 200 Long Beach VA dataset. The total number of instances was 917, covering most of the 918 instances of the nonduplicated CVD dataset.

Before using these datasets, we fixed multiple missing values for the feature “Cholesterol = 0” in the Switzerland and Long Beach VA datasets. Because of multiple missing values in both datasets, we only used the Cleveland and Hungarian datasets to examine the restructuring of trees when consolidating one tree generated from a dataset integrated from both datasets. However, we investigated the traces of all trees hidden in the tree generated from all 918 instances.

In this phase, we used the C4.5 or J48 algorithms from WEKA [31] to discover trees from the 302 instances of the Cleveland dataset and 293 instances of the Hungarian dataset. All components in both tree structures were compared, in addition to the specifics of each tree and the similarities and differences between them.

We joined both datasets to develop an integrated dataset called the C–H dataset. Subsequently, we used the C4.5 algorithm to discover trees from the combined 595 instances of the C–H dataset and 918 instances of the entire CVD dataset. Both integrated datasets were used to examine how the Cleveland and Hungarian components were arranged in the integrated datasets.

The parameters of the C4.5 algorithm for all trees must be set to the same environment for comparison. The default parameters from WEKA are optimal for general-purpose use. However, datasets with significantly different sizes must not use the same parameters for the minimum number of instances per leaf (minNumObj) for comparison purposes. For datasets with the same minNumObj, the tree from the large dataset will be larger and more complex than the tree from the small dataset. The trees from differently sized datasets with the same minNumObj have different complexities of structures that are difficult to compare. Therefore, we cannot objectively discuss the changes in tree structures without validating that the tree structures have similar complexities for comparison purposes.

In this study, we propose a heuristic validation method using the least PTS for tree comparison. The complexity of a tree structure can be viewed as the ratio of leaf nodes to all nodes. Because the number of leaf nodes is n and the number of all nodes is m, we found that from the principle of numerous experiments, 2n > m always holds. However, trees with superior complexity have high values of 2n − m, the more prodigality for the tree structure give more values of 2n – m. Thus, we used the least PTS to validate the complexity of tree structures for comparison purposes. If multiple minNumObjs have the least PTS, we select the minNumObj with the optimal accuracy for the tree structure.

Direct experiments can identify the least PTS from two to any number. Thus, we employed a simple hill-climbing method to determine the least PTS.

The tree structure may be extremely complex if the least PTS is two, the complex tree as this case represents a conflict of interest with the descriptive task. Thus, we selected the minimum size of the tree structure as the least PTS.

For the 918 records of the dataset, we reported that minNumObj = 2–40 is the range of the least PTS of all trees. We selected and demonstrated the minNumObj of each dataset from the experimental data. We used WEKA to discover trees from each dataset at minNumObj = 2 (default), 3, 4, 5, 6, 7, 8, 9, 10, 11, 12, 13, 14, 15, 20, 25, 30, 35, and 40. Then, we selected the minNumObj yielding the least PTS with the optimal accuracy to obtain a compact tree with good structural detail and accuracy.

The results of this phase are provided in Results.

### Phase II: constructing the descriptive forest

Phase I analyzes the presence of trees in the integrated dataset. Thus, the integrated dataset must reveal trees suitable for the dataset’s descriptive tasks. In this phase, we proposed a novel idea to combine trees to form a descriptive forest for the descriptive tasks. We first discovered the main topics or primary features from the integrated dataset. Next, each main topic was considered as the boundary node acting as the root node of a new tree developed using the C4.5 algorithm with the least PTS. Finally, all trees were joined to form the descriptive forest.

This phase comprises three tasks: Task 1 was discovering the main topics from the integrated dataset using the association-rule tree, Task 2 was constructing the tree from each main topic, and Task 3 was constructing the descriptive forest. All the tasks are detailed below.

#### Task 1: Discovering the main topics from the integrated dataset using the association-rule tree

The association-rule tree [25] is a technique for plotting a tree in an interesting space, with support and confidence on the X and Y axes, respectively. The tree has a root node that satisfies the domain rule ∅ ⇒ A, where A is any itemset the user needs to discover related items in the left orderly rule, the related rules. All related rules are discovered using the slope of interestingness developed from the principle of the “profitability-of-interestingness measure” [26]. The rules exhibit increasing rates of confidence rather than decreasing rates of support such that the rules have a relationship with the orderly domain rule, which is a measure that can be transformed to the slope of interestingness to be plotted in the interestingness space.

In this study, we set the domain rule as ∅ ⇒ {Heart Disease = Yes}. Furthermore, we discretized the CVD dataset to numeric features using the supervised filter in WEKA [31]. After generating all the class association rules (CARs) [32] from the dataset using WEKA, we only selected results where the CAR {Heart Disease = Yes} applies to the association-rule tree [25] to discover all the related rules of the domain rule. The itemsets on the left of these related rules are the main topics or primary features related to {Heart Disease = Yes}. Each main topic is a root node connected by a C4.5 tree with the least PTS, as detailed in the next task.

#### Task 2: Constructing the tree from each main topic

The left itemsets of all rules discovered in Task 1 represent the main topics related to {Heart Disease = Yes}. Each itemset is the criterion, or boundary, to select related instances in the CVD dataset without discretization. Thus, each itemset is the root node, or boundary node, to be connected by the C4.5 tree with the least PTS discovered from the related dataset. Because these trees are designed for descriptive tasks, they should be compact in size. Moreover, all trees should have similar complexities for their combined use. To generalize the tree structures for their use as a descriptive forest, we choose the least PTS as a validation policy of the heuristics method.

All trees are constructed using WEKA, with the least PTS selected by experiments where the minNumObj = 2, 3, 4, 5, 6, 7, 8, 9, 10, 11, 12, 13, 14, 15, 20, 25, 30, 35, and 40. The TPS is calculated from the tree structure 2n − m, where n = number of leaf nodes and m = number of all nodes. The minNumObj yielding the least PTS with the optimal accuracy is selected. This task sets the training and test set based on the WEKA parameter in 10-fold cross-validation.

#### Task 3: Constructing the descriptive forest

All the trees from Task 2 are joined to form the descriptive forest. Trees with a boundary node acting as a root node, not in any subset or superset of others, are independent trees. However, trees with a boundary node that is a subset or superset of others are dependent trees. All trees are used for voting and describing (Table 2).

**Table 2:** Example of using a descriptive forest

Instance ID	Boundary nodes (or root node) of trees in the descriptive forest				
	{A}	{B}	{C}	{C,D}	{C,E}
001	Yes	-	Yes	Yes	No
002	Yes	No	Yes	-	-
N	-	Yes	Yes	-	No

In Table 2, the left itemsets of rules discovered from the association-rule tree are the root nodes, or boundary nodes, for connecting the C4.5 tree to its related dataset. The featured items are A, B, C, D, and E. The boundary nodes of the independent trees are {A} and {B}, while those of the dependent trees are {C}, {C, D}, and {C, E}. This example dataset has N instances.

The trees with boundary nodes {A} and {B} can be used by voting or freely describing. The trees with boundary nodes {C}, {C, D}, and {C, E} have overlapping datasets because related instances with feature items were selected as boundary nodes. Following the antimonotone principle [33], the datasets of trees constructed by root nodes {C, D} and {C, E} are subsets of the dataset of trees with root node {C}, while the {C, D} and {C, E} nodes are a superset of the {C} node. Thus, the use of these dependent trees may be duplicated because there is bias in the voting and describing. Instances with a feature of any boundary node of dependent trees must be considered using a method for avoiding bias. First, the trees constructed by the superset nodes are used. If an instance has any features of these trees, the trees are used for voting and describing, while trees constructed by a subset node are not used. If the instance has no features of these trees, trees with a subset node are used for voting and describing.

For example, in Table 2, Instance 001 is used for voting and describing by the {A}-tree, where {A} is the root node of the tree, {C, D}-tree, and {C, E}-tree. The {C}-tree is not used because of the duplicated bias with the {C, D}-tree and {C, E}-tree. Thus, we can describe the CVD risks of Instance 001, as detailed in the {A}-tree and {C, D}-tree, and predict that Instance 001 has {Heart Disease = Yes} because the number voting for Yes = 2 is more than that for No = 1.

Instance 002 is used for voting and describing by the {A}-tree, {B}-tree, and {C}-tree. The {C}-tree is used because this instance is not matched with the {C, D}-tree and {C, E}-tree. Thus, we can describe the CVD risk of Instance 002, as detailed in the {A}-tree and {C}-tree, and predict that Instance 002 has {Heart Disease = Yes} because the number voting for Yes = 2 is more than that for No = 1.

Instance N is used for voting and describing by the {B}-tree and {C, E}-tree. The {C}-tree is not used because of the duplicated bias with the {C, E}-tree. Thus, we can describe the CVD risk of Instance N, as detailed in the {B}-tree, and predict that Instance N has {Heart Disease = No} because the number voting for Yes = 1 equals that for No = 1. In this case, we make predictions using the tree with the optimal F-measure, as precision and recall are related to descriptive quality.

Where no tree matches the instance, the instance is described as having no risk from the primary features discovered from the dataset. Thus, we must predict that this instance also has no risk.

The characteristics of the descriptive forest are analyzed and compared with a single C4.5 tree, as detailed in Phase III. Moreover, the suitability and quality of the descriptive forest are detailed in Phase IV.

### Phase III: comparing each tree structure in the descriptive forest with a single C4.5 tree

In this phase, a single C4.5 tree with the least PTS is constructed for comparison with each tree in the descriptive forest. The single C4.5 tree is constructed by WEKA, using the same method to select the minNumObj of trees in Phase II, Task 2. The parameters used to compare the tree structures are the size, depth, and similar and different components of the trees.

The results are used to examine the reasonability of consolidating a tree to a single C4.5 tree and examine the effects of the disappearance of components from trees or the bias from new components in a single C4.5 tree.

### Phase IV: comparing the usability of the descriptive forest and a single C4.5 tree

Here, the qualitative and quantitative aspects of the descriptive tasks of a single C4.5 tree and the descriptive forest are compared. This phase comprises two sections: Section I compares a qualitative descriptive task between a single C4.5 tree and the descriptive forest, and Section II compares a quantitative descriptive task between a single C4.5 tree and the descriptive forest.

#### Section I: Comparing a qualitative descriptive task between a single C4.5 tree and the descriptive forest

Here, instances with simple and complex cases are selected to describe a single C4.5 tree and the descriptive forest. Subsequently, the quality of descriptive tasks is investigated using both tools.

#### Section II: Comparing a quantitative descriptive task between a single C4.5 tree and the descriptive forest

Here, a quantitative descriptive task between a single C4.5 tree and the descriptive forest is compared. The descriptive task can be considered the coverage and correctness of the explanation and the accuracy, precision, and recall of the dataset description.

The training and test datasets for the single C4.5 tree comprise the entire dataset, i.e., the 918 instances of the CVD, because we are measuring the quality of explanations on the considered dataset and not on the training set, test set, or new data.

The test dataset for all the trees of the descriptive forest comprises the 918 instances of the CVD dataset. However, the training dataset for each tree in the descriptive dataset is used only for related instances of the CVD dataset, while instances matching features at the boundary nodes are selected for the training dataset.

Accuracy, precision, and recall can sufficiently measure the quality (coverage and correctness) of the descriptive task on the considered dataset but not on new data (i.e., predictive tasks).

### Phase V: applying the proposed method to a bigger dataset

This phase fosters the acceptability of the explanations using the proposed method with a bigger dataset. We chose 253,680 records with 22 features of imbalanced classes of the heart disease health–indicators dataset [28] that has only 23,893 heart disease records.

This phase shows that a descriptive forest without the least PTS can be used for classification by comparing it with a single C4.5 tree without the least PTS. However, the numerous nodes of the tree structure are difficult to use for descriptive tasks.

Hence, we repeat Phase II with this larger dataset. Afterwards, we compare the results of the descriptive forest and a single C4.5 tree.

## Results

The results of the five phases are detailed as follows.

### Results of Phase I: existence of many tree structures generated from an integrated CVD data set

The phase results are obtained from 302 instances of the Cleveland dataset, 293 instances of the Hungarian dataset, 595 instances of the C–H dataset, and 918 instances of the entire CVD dataset. All datasets have 11 features and one class. The features are age, sex, chest pain type (ChestPainType), resting blood pressure (RestingBP), cholesterol, fasting blood sugar (FastingBS), resting ECG (RestingECG), maximum heart rate (MaxHR), exercise-induced angina (ExerciseAngina), old peak (Oldpeak), and ST slope (ST_Slope). The class is heart disease, in a binary “Yes” or “no.”

Each tree is developed by the C4.5 tree from WEKA, or J4.8, with the least PTS. The minNumObjs of trees constructed by the Cleveland, Hungarian, and C–H datasets are 40, 11, and 20. The least PTS of trees constructed using all the CVD datasets is 14. All the experiments are listed in Table 3.

**Table 3:** Least PTS of each tree constructed by WEKA

minNumObj	Cleveland dataset				Hungarian dataset				C–H dataset				All the CVD datasets			
	acc	LN	size	PTS	acc	LN	size	PTS	acc	LN	size	PTS	acc	LN	size	PTS
2	72.52	40	68	12	90.79	3	4	*2*	86.39	26	43	9	85.19	34	60	8
3	71.85	33	56	10	90.44	3	4	*2*	85.04	26	42	10	85.73	17	30	4
4	71.85	13	21	5	92.49	3	4	*2*	85.04	17	27	7	85.62	16	28	4
5	68.87	12	19	5	92.15	3	4	*2*	85.55	17	27	7	85.19	16	28	4
6	69.87	6	9	3	90.79	3	4	*2*	82.86	12	21	3	84.10	16	28	4
7	71.19	6	9	3	91.47	3	4	*2*	83.53	8	14	*2*	84.10	14	24	4
8	73.18	6	9	3	91.81	3	4	*2*	83.53	8	14	*2*	83.55	16	28	4
9	73.18	6	9	3	92.49	3	4	*2*	83.53	8	14	*2*	83.66	12	22	*2*
10	72.52	6	9	3	92.49	3	4	*2*	83.36	8	14	*2*	83.33	12	22	*2*
**11**	72.52	6	9	3	**93.17**	5	8	*2*	83.53	8	14	*2*	83.12	12	22	*2*
12	70.86	6	10	2	93.17	3	4	*2*	83.70	7	12	*2*	82.68	12	20	4
13	70.86	6	10	2	93.17	3	4	*2*	83.70	7	12	*2*	83.22	12	20	4
**14**	70.53	6	10	2	93.17	3	4	*2*	83.70	5	8	*2*	**83.88**	10	18	*2*
15	69.87	6	10	2	93.17	3	4	*2*	83.70	5	8	*2*	83.55	10	18	*2*
**20**	70.20	6	10	2	93.17	3	4	*2*	**84.20**	5	8	*2*	83.12	9	16	*2*
25	70.53	5	9	*1*	93.17	3	4	*2*	83.70	5	8	*2*	83.44	7	12	*2*
30	71.52	5	8	2	93.17	3	4	*2*	81.68	5	8	*2*	82.35	6	10	*2*
35	70.86	4	7	*1*	93.17	3	4	*2*	80.84	4	6	*2*	81.05	6	10	*2*
**40**	**71.52**	4	7	*1*	93.17	3	4	*2*	80.84	4	6	*2*	81.05	4	6	*2*

Note: *Acc* accuracy, *LN* Number of Leaf Nodes, *Size* tree size, and *PTS* Prodigality for the Tree Structure

From Table 3, all minNumObjs yield good accuracy, similar to the accuracy from the default minNumObj (2). However, the size of the tree structure is significantly reduced. The tree size with the least PTS in the Cleveland dataset is reduced from 68 to 7. Furthermore, the tree size with the least PTS in the C–H dataset is reduced from 43 to 8, and the tree size with the least PTS in all the CVD datasets is reduced from 60 to 18. However, the tree size with the least PTS in the Hungarian dataset is 8, greater than 4 because it is the same as the least PTS while exhibiting better accuracy.

**Fig. 1:**
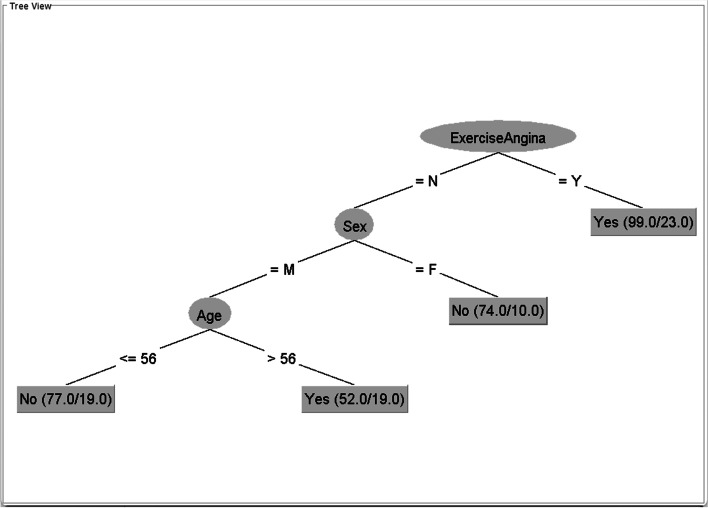
Tree generated from the Cleveland dataset with minNumObj = 40 This figure is generated by the WEKA software

**Fig. 2:**
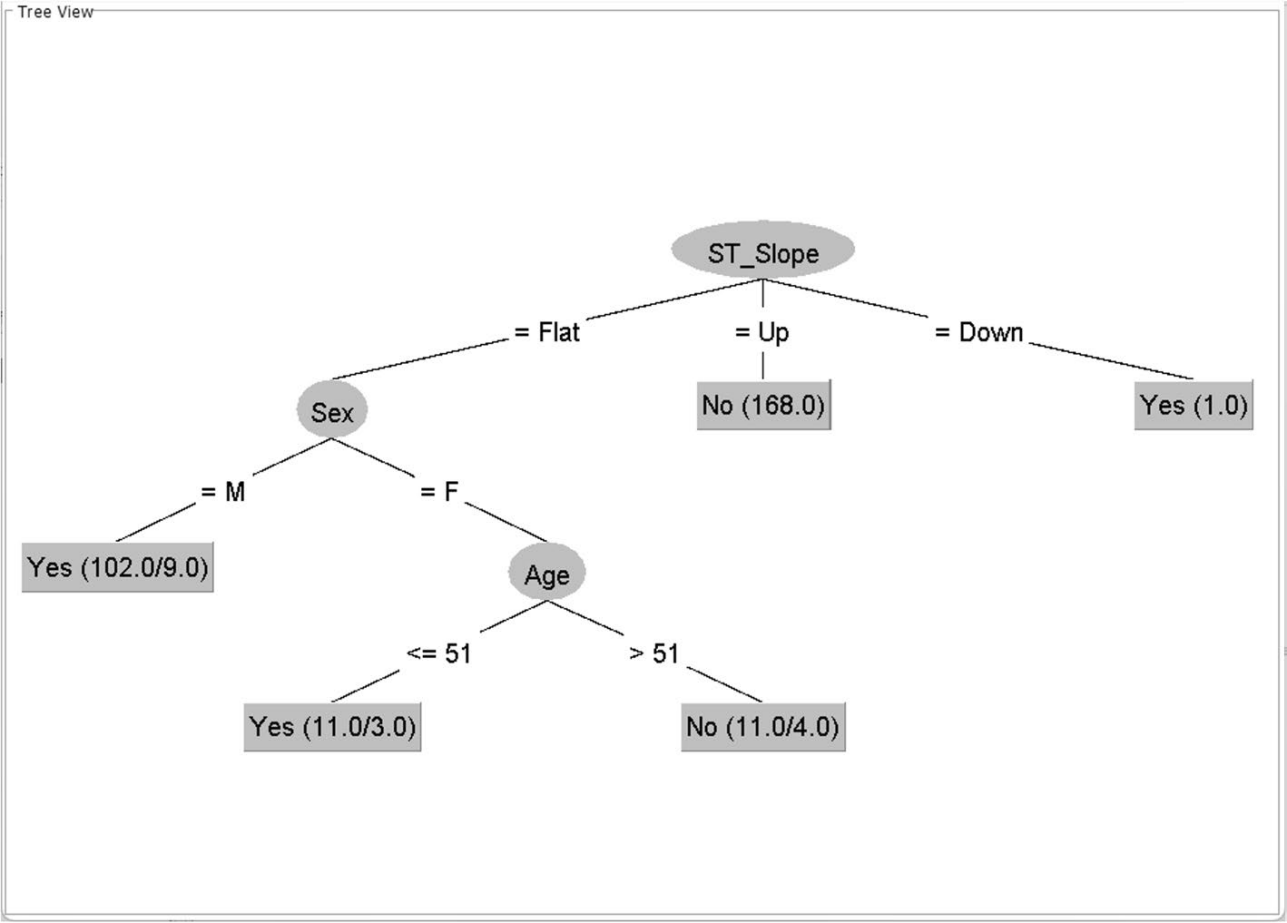
Tree generated from the Hungarian dataset with minNumObj = 11 This figure is generated by the WEKA software

**Fig. 3:**
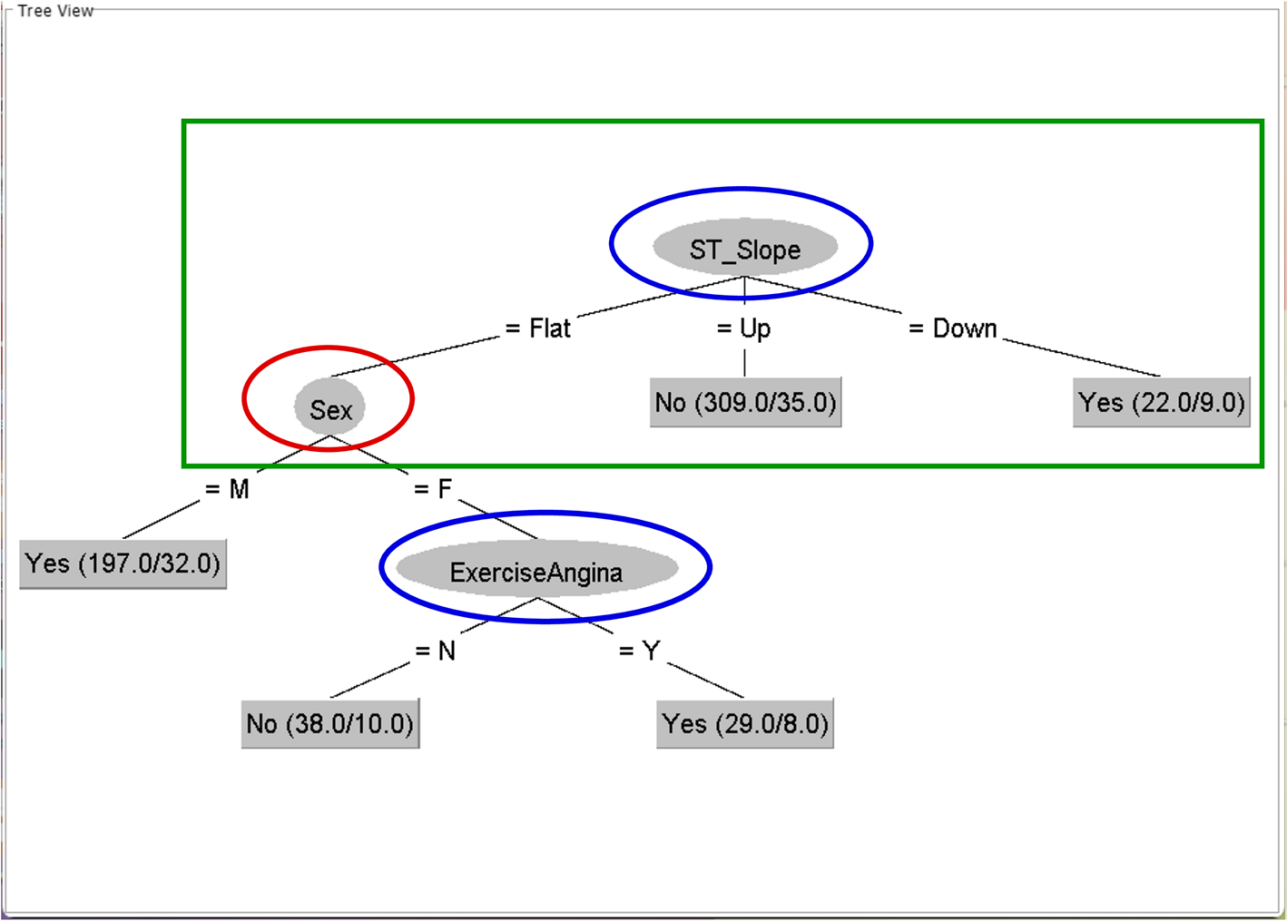
Tree generated from the C–H dataset with minNumObj = 20 This figure is generated by the WEKA software

**Fig. 4:**
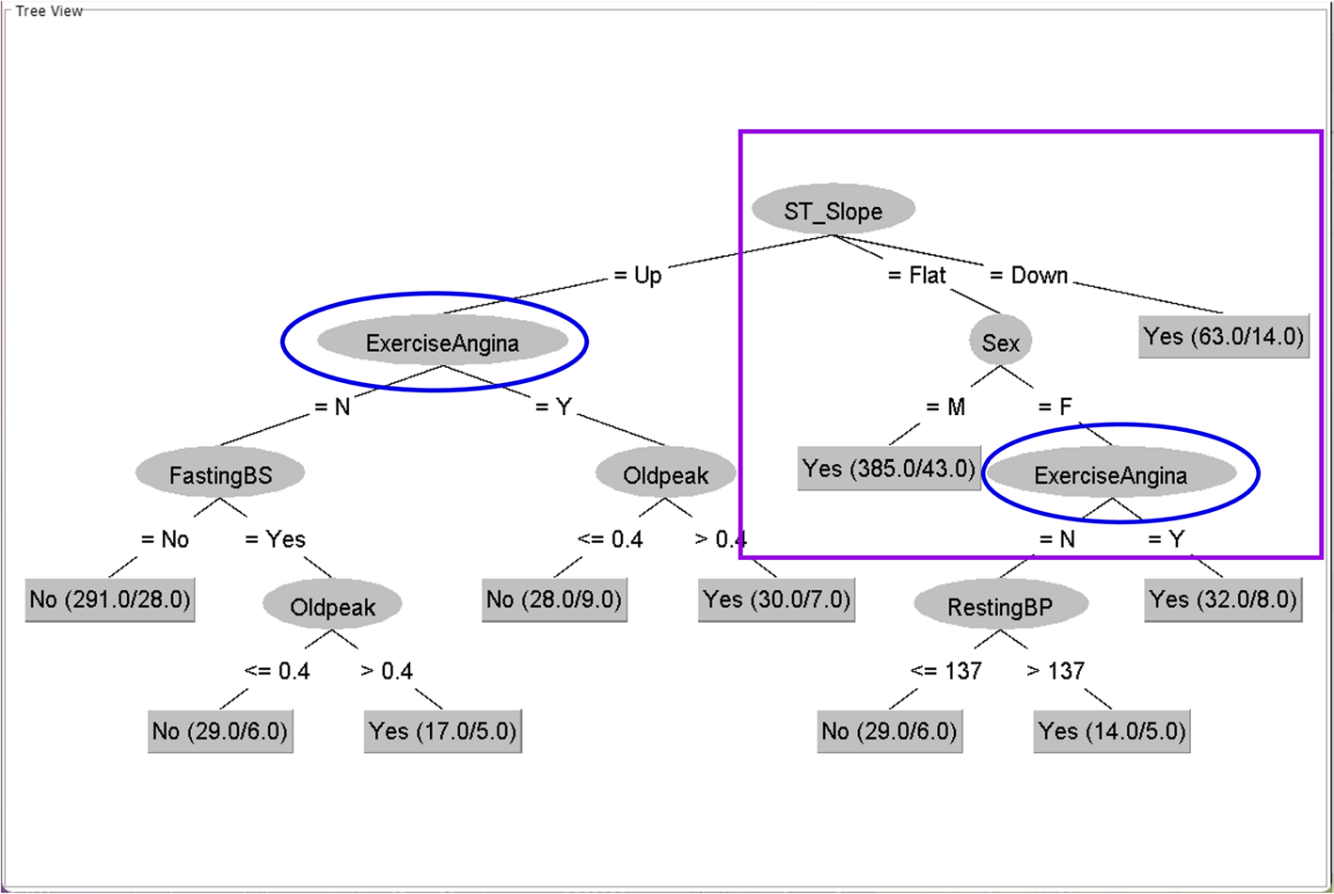
Tree generated from all instances of CVD datasets with minNumObj = 14 This figure is generated by the WEKA software

Figures 1, 2, 3 and 4 show the trees generated from these datasets with the least PTSs.

The trees in Figs. 1 and 2 have different structures. The tree from the Cleveland dataset comprises exercise-induced angina (root node), sex, age, and leaf nodes. The tree from the Hungarian dataset comprises ST slope (root node), sex, age, and leaf nodes. We will examine how these components are restructured in the integrated datasets (the C–H dataset and all CVD datasets), as shown in Figs. 3 and 4.

The part of the tree in the green rectangle in Fig. 3 is the tree structure from the Hungarian dataset. The root node and ST slope of the C–H dataset tree are the same as in the Hungarian dataset. Furthermore, the root node from the Cleveland dataset is still in the C–H dataset tree. The Cleveland dataset is larger than the Hungarian dataset. However, the root node of the Cleveland dataset is of reduced importance because of its low position in the C–H dataset tree. The blue circles show the new positions of the root nodes from the Cleveland and Hungarian datasets.

This representation indicates that these were the primary topics in the datasets before being consolidated into the integrated dataset and competing to be the winning root nodes in the integrated dataset. The losing main topic is of reduced importance in the integrated dataset, while the other topics that work well with the winner are the main topics it promotes.

The root node of the dataset is the main topic related to the classes. Therefore, different datasets with different main topics are presented as root nodes. The greedy algorithm performs tree induction to select the root node from the most important features in the dataset. Thus, the integrated dataset is biased in selecting only one main topic, while the other topics become of reduced importance because of their low levels in the tree structure. Some main topics may even disappear.

For example, the age feature node from the Cleveland and Hungarian datasets disappears in the C–H dataset tree because the age features from different datasets are in different environments that provide different roles in the age features. Thus, the roles of these age features are consolidated and disappear in the integrated dataset.

The sex node positions are the same in all three trees, as subtree level 1 is next to the root node. This representation of the sex feature contrasts that of the age feature. This is because the sex feature in different environments may have the same role in all datasets or work together with the cofeatures or secondary features that are imperative in all three trees.

In Fig. 4, the winning main topic, the ST slope, is still the root node, and part of the tree is inherited from Figs. 2 and 3 (purple rectangle). Nevertheless, the other main topic in the blue oval, exercise-induced angina, still has a role in this CVD dataset tree. The roles of exercise-induced angina are fragmented in numerous positions in the tree. These fragmented roles can be observed in the Oldpeak feature. Thus, we hypothesize that the Oldpeak feature may be the main topic that is difficult to discover from the integrated dataset. The bias from the root node, the main winning topic, reduces the importance of the other topics and may promote certain cofeatures of the root node.

The results of Phase I indicate that various CVD datasets may have different trees, each with its main topic. However, in the integrated dataset, these main topics compete with the root node, whose bias can reduce or promote other topics to work together. In addition, each dataset may have already been collected from different environments so that each dataset can have more than one main topic. Consequently, the main topics may outnumber the datasets.

In contrast, we propose a method to discover a subdataset with its main topic adopted from the integrated dataset. Each tree of these subdatasets can discover other topics to work together with the main topic at the root node. This approach is suitable for descriptive tasks. The results of our proposed method, the descriptive forest, are detailed next.

### Results of Phase II: descriptive forest discovered from the CVD dataset

The results in this phase are performed by three tasks, as follows.

#### Results of Task 1

The numeric features of the CVD dataset are discretized for the association-rule discovery using WEKA [31]. Afterward, two features, resting blood pressure and cholesterol, are filtered out because they have only one feature’s value. Next, the parameters of the a priori algorithm are set to support = 0 and confidence = 0, and “discover only CARs” is set to “discover all CARs.”

We found 60,349 CARs with 30,306 positive CARs, the CARs with Class {Heart Disease = Yes}. We selected the positive CARs to calculate the slope of interestingness using the association-rule tree [25]. We found only eight rules, except the domain rule, presented as “antecedent itemsets ⇒ {Heart Disease = Yes}” in Table 4.

**Table 4:** Rules discovered from the CVD Dataset using the association-rule-tree discovery

Rules: antecedent itemsets ⇒ {Heart Disease = Yes}	Support	Confidence
Domain Rule: ∅ ⇒ {Heart Disease = Yes}	0.55	0.55
{Sex = M} ⇒ {Heart Disease = Yes}	0.50	0.63
{Sex = M, ChestPainType = ASY} ⇒ {Heart Disease = Yes}	0.38	0.83
{Sex = M, ExerciseAngina = Y} ⇒ {Heart Disease = Yes}	0.31	0.88
{Sex = M, ST_Slope = Flat} ⇒ {Heart Disease = Yes}	0.37	0.89
{ChestPainType = ASY} ⇒ {Heart Disease = Yes}	0.43	0.79
{ExerciseAngina = Y} ⇒ {Heart Disease = Yes}	0.34	0.85
{Oldpeak > 0.85} ⇒ {Heart Disease = Yes}	0.36	0.78
{ST_Slope = Flat} ⇒ {Heart Disease = Yes}	0.42	0.83

**Fig. 5:**
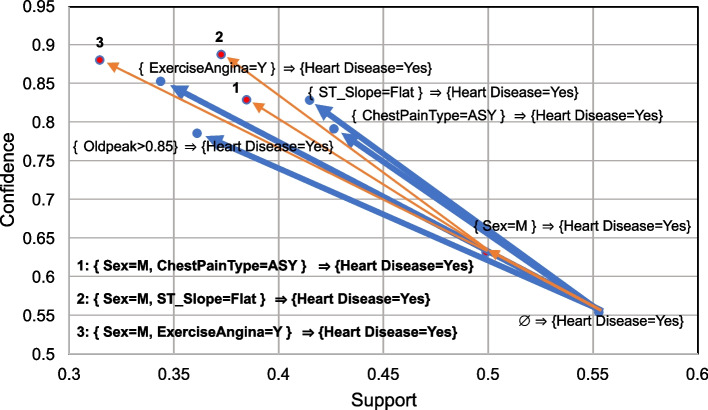
Association-rule tree discovered from the CVD dataset

Table 4 shows that all the discovered rules exhibit support and confidence. All rules can be plotted in the interestingness area, with the X-axis as the support and the Y-axis as the confidence. The strong relationship between nodes measured by the slope of interestingness can be plotted as the edges. Figure 5 shows the discovered association-rule tree.

Figure 5 shows that the eight rules, except the domain rule, are in two subgroups. The first subgroup has four independent rules, with only one member in the left itemset of each rule: {ExerciseAngina = Y} ⇒ {Heart Disease = Yes}, {ST_Slope = Flat} ⇒ {Heart Disease = Yes}, {ChestPainType = ASY} ⇒ {Heart Disease = Yes}, and {Oldpeak > 0.85} ⇒ {Heart Disease = Yes}.

The main topics in the first subgroup are the left itemset of the independent rules. These independent topics are {ExerciseAngina = Y}, {ST_Slope = Flat}, {ChestPainType = ASY}, and {Oldpeak > 0.85}.

The other subgroup has four dependent rules, and three rules have left itemsets that are a superset of {Sex = M}, plotted by the red nodes and red text. The rules {Sex = M, ChestPainType = ASY} ⇒ {Heart Disease = Yes}, {Sex = M, ST_Slope = Flat} ⇒ {Heart Disease = Yes}, and {Sex = M, ExerciseAngina = Y} ⇒ {Heart Disease = Yes} have a strong relationship with the rule {Sex = M} ⇒ {Heart Disease = Yes}.

The main topics in the second subgroup are the left itemsets of the dependent rules. These dependent topics are {Sex = M} and its superset of {Sex = M, ChestPainType = ASY}, {Sex = M, ST_Slope = Flat}, and {Sex = M, ExerciseAngina = Y}.

All rules have the main topics that are the left itemsets of rules. We use each main topic to select related instances, with each instance having features as the main topic of the CVD dataset. Each main topic and related dataset are constructed in the C4.5 tree with the least PTS, as shown in the following results.

#### Results of Task 2

All rules discover a previous task with a left itemset that has features related to the class {Heart Disease = Yes}. All left itemsets of the rules are defined as the main topics. These main topics can select related instances from the CVD dataset by matching each main topic to each instance of the dataset. For example, the fourth instance has Oldpeak = 1.5. Thus, this instance matches the main topic {Oldpeak > 0.85}. The number of instances matching each main topic is shown in Table 5.

**Table 5:** Number of instances matching each main topic

Main Topic	Count	% of the CVD Dataset
ChestPainType = ASY	496	54.03
ExerciseAngina = Y	371	40.41
Oldpeak > 0.85	423	46.08
ST_Slope = Flat	460	50.11
Sex = M	**725**	78.98
Sex = M, ChestPainType = ASY	426	46.41
Sex = M, ExerciseAngina = Y	328	35.73
Sex = M, ST_Slope = Flat	385	41.94

From Table 5, all subsets of the CVD dataset have sizes of approximately 35%–80% of the dataset. Notably, the count of the main topic {Sex = M} is presented as 725 or 78.98%, but this main topic is one of the subgroups of the dependent rules. Only the role of {Sex = M} matches 174 instances or 18.95%.

Each main topic and its related instances (Table 5) are constructed as a C4.5 tree with the least PTS. All eight trees are summarized by transforming them to If–Then rules (Table 6) for effective and concise representation.

**Table 6:** The If–Then rules transformed from the C4.5 Trees constructed by the main topics

Main Topic	The minNumObj of Leaf Nodes at the Least PTS	The If–Then rules transformed from the C4.5 trees
ChestPainType = ASY	40	If {ChestPainType = ASY}, then consider {ST_Slope} (if {ST_Slope = Down}, then {Heart Disease = Yes}, if {ST_Slope = Flat}, then {Heart Disease = Yes}, if {ST_Slope = Up} then consider {Oldpeak} ( if {Oldpeak ≤ 0.4} then {Heart Disease = No}, if {Oldpeak > 0.4} then {Heart Disease = Yes}))
ExerciseAngina = Y	15	If {ExerciseAngina = Y} then consider {MaxHR} ( if {MaxHR ≤ 150} then {Heart Disease = Yes}, if {MaxHR > 150} then consider {Oldpeak}( if {Oldpeak ≤ 0.8} then {Heart Disease = No}, if {Oldpeak > 0.8} then {Heart Disease = Yes}))
Oldpeak > 0.85	9	If {Oldpeak > 0.85} then consider {MaxHR} ( if {MaxHR ≤ 150 then consider {Sex} ( if{Sex = M} then {Heart Disease = Yes}, if {Sex = F} then consider {ExerciseAngina} ( if {ExcerciseAngina = N} then {Heart Disease = No}, if { ExcerciseAngina = Y} then {Heart Disease = Yes})), if {MaxHR > 150} then consider {Oldpeak} ( if {Oldpeak ≤ 2.4} then consider {ExerciseAngina} (if {ExcerciseAngina = N} then {Heart Disease = No}, if {ExcerciseAngina = Y} then {Heart Disease = Yes}), if {Oldpeak > 2.4} then {Heart Disease = Yes}))
ST_Slope = Flat	5	If {ST_Slope = Flat} then consider {Sex} ( if {Sex = M} then {Heart Disease = Yes}, if {Sex = F} then consider {FastingBS} ( if {FastingBS = Yes} then {Heart Disease = Yes}, if { FastingBS = No} then consider {ExerciseAngina} (if { ExcerciseAngina = Y} then {Heart Disease = Yes}, if { ExcerciseAngina = N} then consider {RestingBP} ( if {RestingBP ≤ 146} then {Heart Disease = No}, if {RestingBP > 146} then {Heart Disease = Yes}))))
Sex = M, ChestPainType = ASY	35	If {Sex = M, ChestPainType = ASY} then consider {ST_Slope} (if {ST_Slope = Down} then {Heart Disease = Yes}, if {ST_Slope = Flat} then {Heart Disease = Yes}, if {ST_Slope = Up} then consider {OldPeak} ( if {OldPeak ≤ 0.4} then {Heart Disease = No}, if {OldPeak > 0.4} then {Heart Disease = Yes}))
Sex = M, ExerciseAngina = Y	7	If {Sex = M, ExerciseAngina = Y} then consider {MaxHR} ( if {MaxHR ≤ 150} then {Heart Disease = Yes}, if {MaxHR > 150} then consider {FastingBS} ( if {FastingBS = Yes} then {Heart Disease = Yes}, if {FastingBS = No} then consider {ST_Slope} (if {ST_Slope = Down} then {Heart Disease = No}, if {ST_Slope = Flat} then {Heart Disease = No}, if {ST_Slope = Up} then consider {MaxHR}( if {MaxHR ≤ 162} then {Heart Disease = Yes}, if {MaxHR > 162} then {Heart Disease = No}))))
Sex = M, ST_Slope = Flat	2	If { Sex = M, ST_Slope = Flat} then consider {ChestPainType} ( if {ChestPainType = ASY} then {Heart Disease = Yes}, if {ChestPainType = NAP} then consider {Age} ( if {Age ≤ 44} then {Heart Disease = No}, if {Age > 44} then {Heart Disease = Yes}), if {ChestPainType = ATA} then consider {Cholesterol} ( if {Cholesterol > 245} then {Heart Disease = Yes}, if {Choleserol ≤ 245} then consider {MaxHR}( if {MaxHR ≤ 130} then {Heart Disease = Yes}, if {MaxHR > 130} then {Heart Disease = No})), if {ChestPainType = TA} then consider RestingECG( if {Resting ECG = Normal} then {Heart Disease = Yes}, {Resting ECG = ST} then {Heart Disease = Yes}, if {RestinfECG = LVH} then consider {Cholesterol} (if {Cholesterol ≤ 258} then {Heart Disease = No}, if {Choleserol > 258} then {Heart Disease = Yes})))
Sex = M	15	If ( {Sex = M}, {ChestPainType ≠ ASY}, {ExerciseAngina ≠ Y}, { ST_Slope ≠ Flat}) then consider {ST_Slope} ( if {ST_Slope = Down} then {Heart Disease = No}, if {ST_ST_slope = up} then consider {OldPeak}( if {OldPeak ≤ 0.4} then {Heart Disease = No},}( if {OldPeak > 0.4} then consider { FastingBS}( if {FastingBS = No} then {Heart Disease = No}, if {FastingBS = Yes} then {Heart Disease = Yes})))

**Fig. 6:**
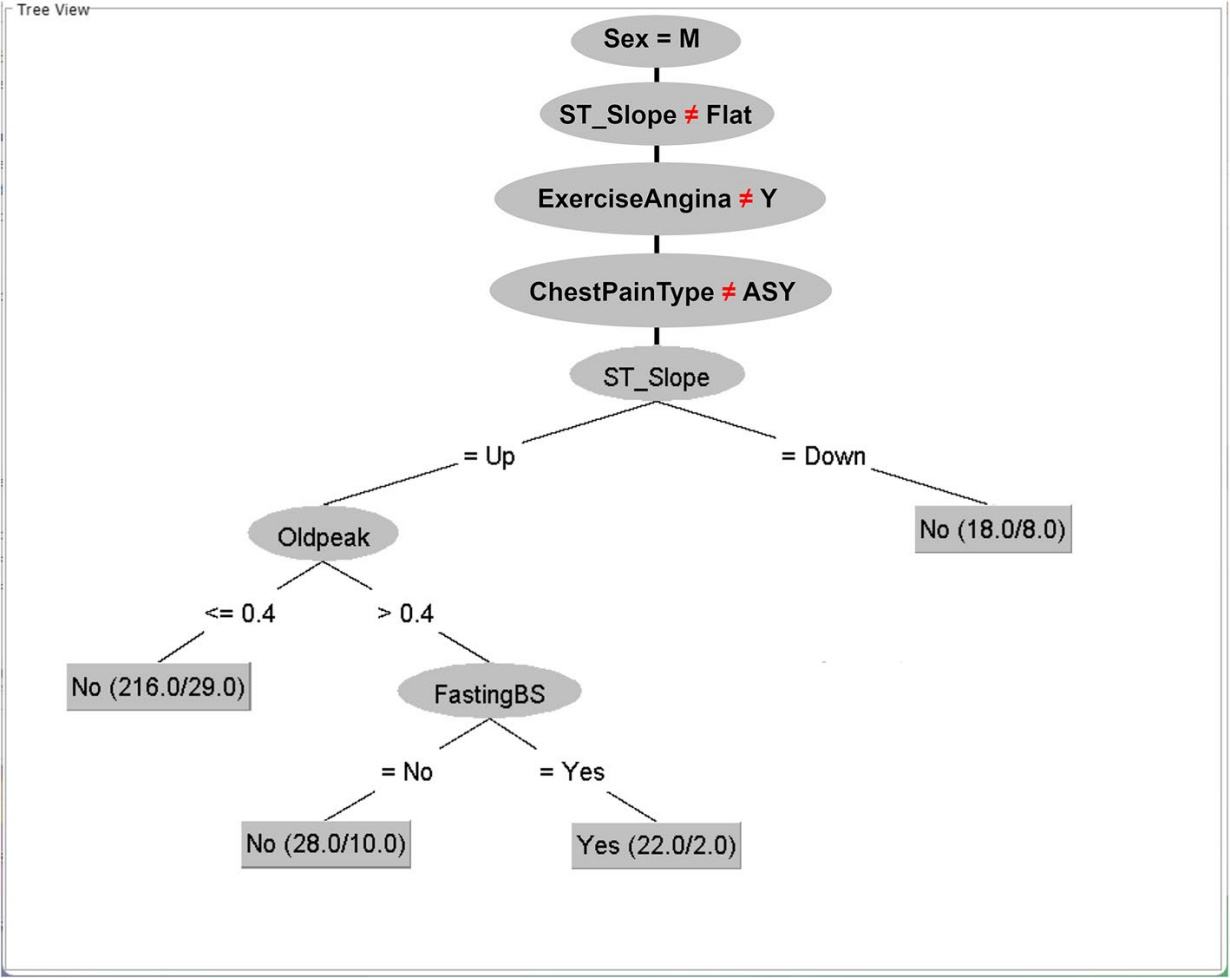
The {Sex = M}-tree reconstructed for use with its dependent trees This figure is generated by the WEKA software

From Table 6, all C4.5 trees constructed by the main topics are transformed into If–Then rules. However, dependent trees are used together in the descriptive forest. The three trees related by the {Sex = M} topic are used first. If these trees do not match an instance, then a tree constructed using only the {Sex = M} topic is used. Thus, the {Sex = M}-tree must be reconstructed by removing all the nodes and their main-dependent topics and adding the boundary nodes (Fig. 6). This tree is then transformed into the If–Then rule of the {Sex = M}-tree in Table 6. All trees from this task form the descriptive forest, as shown in the following results.

#### Results of Task 3

All the trees from the previous task work together as the descriptive forest. Descriptive tasks can be defined by voting and describing using the descriptive forest.

Voting defines the presence or absence of risk in each instance in the CVD dataset. Thus, the result may be similar or different from the class of instances. However, voting in the same dataset for training and testing, which does not use new instances, is a descriptive data mining task and not a predictive task.

All trees in the descriptive forest are constructed using two subgroups of the main topics, forming independent and dependent subgroups. The use of each independent tree allows voting or describing freely.

However, dependent trees have an order to construct, and some trees may not be used. First, the trees with the maximum number of item members of the root node are used for voting or describing freely. If an instance matches any tree, then voting or describing may be performed by the matched trees, except for trees with the root node, which is a subset of the matched tree nodes. Second, if no tree from the first step is used, then the tree with the root node is used, whose item members in the root node are a subset of the trees in the first step. These first and second steps occur in a loop until no dependent trees remain.

The independent trees are {ExerciseAngina = Y}-tree, {ST_Slope = Flat}-tree, {ChestPainType = ASY}-tree, and {Oldpeak > 0.85}-tree. Conversely, the dependent trees are {Sex = M, ChestPainType = ASY}-tree, {Sex = M, ST_Slope = Flat}-tree, {Sex = M, ExerciseAngina = Y}-tree, and {Sex = M}-tree. All trees are related to the last tree, {Sex = M}-tree, whose item member of the root node is a subset of the item member in the root node of others. Thus, {Sex = M, ChestPainType = ASY}-tree, {Sex = M, ST_Slope = Flat}-tree, and {Sex = M, ExerciseAngina = Y}-tree must be considered before the {Sex = M}-tree. If no dependent trees match this instance, then the instance must be considered by the {Sex = M}-tree.

Two examples (Instances ID 012 and ID 483 of the CVD dataset) of using the descriptive forest are detailed in Tables 7 and 8.

**Table 7:** Examples from the CVD dataset

Features	Instance ID	
	012	483
Age	58	67
Sex	M	M
ChestPainType	ATA	TA
RestingBP	136	142
Cholesterol	164	270
FastingBS	No	Yes
RestingECG	ST	Normal
MaxHR	99	125
ExerciseAngina	Y	N
Oldpeak	2	2.5
ST_Slope	Flat	Up
HeartDisease	Yes	Yes

**Table 8:** Use of the descriptive forest

Trees with the main topic at the root node	ID of Instance	
	012	483
{Oldpeak > 0.85}-tree	TP	TP
{ChestPainType = ASY}-tree	-	-
{ExerciseAngina = Y}-tree	TP	-
{ST_Slope = Flat}-tree	TP	-
{Sex = M}-tree	-	TP
{Sex = M,ChestPainType = ASY}-tree	-	-
{Sex = M,ExerciseAngina = Y}-tree	TP	-
{Sex = M,ST_Slope = Flat}-tree	TP	-
Voting	TP	TP

The CVD dataset with 918 instances is used as a test set in WEKA, with examples presented in Table 7. Subsequently, all the trees in the descriptive forest generated from their related instances are used as the training dataset. The results can be combined, as shown in Table 8.

The instance with ID 012 is defined as “has CVD risks” from voting by five main topics. Among them, three main topics are independent—{Oldpeak > 0.85}, {ExerciseAngina = Y}, and {ST_Slope = Flat}. The other two—{ExerciseAngina = Y} and {ST_Slope = Flat}—are dependent main topics related to {Sex = M}, which promotes heart disease.

The risks of Instance ID 012 from each main topic can be described. From the {Oldpeak > 0.85}-tree, this instance describes the risk assumed by having MaxHR <  = 150 and Sex = M. From {ExerciseAngina = Y}-tree, this instance describes the risk assumed by having MaxHR <  = 150. From {ST_Slope = Flat}-tree, this instance describes the risk assumed by having Sex = M. From the {Sex = M, ExerciseAngina = Y}-tree, this instance describes the risk assumed by having MaxHR <  = 150. From the {Sex = M, ST_Slope = Flat}-tree, this instance describes the risk assumed by having ChestPainType = ATA, Cholesterol <  = 245, and MaxHR <  = 130.

The instance with ID 483 is defined as “has CVD risks” from voting by two main topics: one from the main independent and dependent topics each. The independent main topic is {Oldpeak > 0.85}, and the dependent main topic is {Sex = M}.

The risks of Instance ID 483 from each main topic can be described. From the {Oldpeak > 0.85}-tree, this instance describes the risk assumed by having MaxHR <  = 150 and Sex = M. From the {Sex = M}-tree, this instance describes the risk assumed by having ST_Slope = Up, Oldpeak > 0.4, and FastingBS = Yes.

In the case of equal voting, where the number of positive and negative votes is equal, we treat the result as negative voting because of the precision of the descriptive task.

If an instance matches no trees, then the instance has no risk from any main topics discovered from the CVD dataset.

The quality of the descriptions performed by the descriptive forest is compared with that of a single C4.5 tree, as shown in the results of Phase IV.

Before comparing the quality of descriptions between the descriptive forest and a single C4.5 tree, their tree structures must be compared. The results are shown in the following phase.

### Results of Phase III: comparing the tree structures of trees from the descriptive forest and a single C4.5 tree discovered from the same CVD dataset

The CVD dataset constructs a single C4.5 tree (Fig. 4) with minNumObj = 14, yielding the least PTS with an accuracy of 83.88%. Next, all trees in the descriptive forest are constructed, as shown in Table 7, and transformed into the If–Then rules for the compact representation. The results of these tree structure comparisons are shown in Table 9.

**Table 9:** Comparing the tree structures between trees from the descriptive forest and a single C4.5 tree

	All trees are performed from the CVDs dataset										annotation
The details of comparisons	the single C4.5 tree	the {Oldpeak > 85}-tree	the {ChestPainType = ASY}-tree	the {ExerciseAngina = Y}-tree	the {ST_Slope = Flat}-tree	the {Sex = M}-tree	the {Sex = M, ChestPainType = ASY}-tree	the {Sex = M, ExerciseAngina = Y}-tree	the {Sex = M, ST_Slope = Flat}-tree	the descriptive forest	
1. Tree size	18	11	6	5	9	7	6	10	16	5–16	excluded the boundary nodes
2. Number of leaf nodes	10	6	4	3	5	4	4	6	10	3–10	
3. Tree depth	4	3	2	2	4	3	2	4	3	2–4	excluded the boundary nodes
4. Number of related instances	918	423	496	371	460	**174**^**1**^	426	328	385	843	
5. Occurrence of the Age feature	-	-	-	-	-	-	-	-	1	1	
6. Occurrence of the Sex feature	1	1	-	-	1	b	b	b	b	b,2	b = boundary node
7. Occurrence of the ChestPainType feature	-	-	b	-	-	b	b	-	1	b,1	b = boundary node
8. Occurrence of the RestingBP feature	1	-	-	-	1	-	-	-	-	1	
9. Occurrence of the cholesterol feature	-	-	-	-	-	-	-	-	2	2	
10. Occurrence of the FastingBS feature	1	-	-	-	1	1	-	1	-	3	
11. Occurrence of the RestingECG feature	-	-	-	-	-	-	-	-	1	1	
12. Occurrence of the MaxHR feature	-	1	-	1	-	-	-	2	1	5	
13Occurrence of the ExerciseAngina feature	2	2	-	b	1	b	-	b	-	b,3	b = boundary node
Occurrence of the Oldpeak feature	2	b,1	1	1	-	1	1	-	-	b,5	b = boundary node
15. Occurrence of the ST_Slope feature	r	-	1	-	b	b,1	1	1	b	b,4	r = root node, b = boundary node

Note:^1^Bold text denotes a special case to calculate the number of related instances. The related instances of the {Sex = M}-tree are reduced from 725 to 174 because they work with dependent trees; the related instances of the descriptive forest are 843, while the remaining 75 instances in the CVD dataset can be defined as “has no risk” from the main topics discovered from the CVD dataset

Table 9 shows that the single C4.5 tree is the largest tree constructed from all instances of the CVD dataset but uses only six features. All features are biased by only one root node. While the descriptive forest has many small trees constructed from 19.0% to 54.0% of the CVD dataset, all trees cover 91.8% and use all features in the CVD dataset. All features are biased from the five boundary nodes of the eight main topics. Moreover, the descriptive forest defines the remaining 8.2% of the CVD dataset as “has no risk” from the main topics discovered from the CVD dataset. Thus, the descriptive forest can vote for and describe all instances of the CVD dataset, indicating the suitability of complex descriptive tasks for the entire CVD dataset.

The results of the last phase confirm the descriptive forest’s suitability and quality and are shown next.

### Results of Phase IV: comparing the usability of the descriptive forest and a single C4.5 tree

The results of this phase are separated into two sections: a comparison of the explanations and the correctness and coverage of these explanations.

#### Results of Section I

The descriptive forest’s explanations of ID 012 and ID 483 are described in the results for Task 3. We compare these explanations with those of a single C4.5 tree (Table 10).

**Table 10:** Comparison of explanations yielded by the single C4.5 tree and the descriptive forest

ID	The Single C4.5 Tree		The descriptive forest	
	Prediction	The Explanation	Voting	The Explanation
012	TP	ID 012 is predicted as “Heart Disease = Yes” because **ST_Slope = Flat** and **Sex = M**	TP	ID 012 is defined as “has CVD risks” from the voting by five main topics. The three main topics are independent: **{Oldpeak > 0.85}, {ExerciseAngina = Y}**, and **{ST_Slope = Flat}**. Two main topics, *{ExerciseAngina = Y}* and *{ST_Slope = Flat}*, are dependent main topics related to *{Sex = M}*, which promotes heart disease Moreover, the risks of Instance 012 from each main topic can be described. For the **{Oldpeak > 0.85}-tree**, this instance describes the risk assumed by having **MaxHR < = 150** and **Sex = M**. For the **{ExerciseAngina = Y}-tree**, this instance describes the risk assumed by having **MaxHR < = 150**. For the **{ST_Slope = Flat}-tree**, this instance describes the risk assumed by having **Sex = M**. For the {*Sex = M, ExerciseAngina = Y}-tree*, this instance describes the risk assumed by having *MaxHR < = 150*. For the *{Sex = M, ST_Slope = Flat}-tree*, this instance describes the risk assumed by having *ChestPainType = ATA, Cholesterol < = 245*, and *MaxHR < = 130*
483	TP	ID 483 is predicted as “Heart Disease = Yes” because **ST_Slope = Up, ExerciseAngina = No, FastingBS = Yes**, and **Oldpeak > 0.4**	TP	ID 483 is defined as “has CVD risks” from the voting by two main topics, **{Oldpeak > 0.85}** and *{Sex = M}*. {Sex = M} is from a dependent tree that includes *{ExerciseAngina ≠ Yes}, {ST_Slope ≠ Y}*, and *{ChestPainType ≠ ASY}* The risks of Instance 483 from each main topic can be described. For the **{Oldpeak > 0.85}-tree**, this instance describes the risk assumed by having **MaxHR < = 150** and **Sex = M**. For the *{Sex = M}-tree*, this instance describes the risk assumed by having *ST_Slope = Up, Oldpeak > 0.4*, and *FastingBS = Yes*

Note: An bold text is a feature from an independent tree or one tree, and a italic text is a feature from a dependent tree

A single C4.5 tree yields a short explanation of ID 012 that covers 385 instances of the CVD dataset, with false positives accounting for 43 instances. This accuracy is good, but the descriptive task is biased from the root node ST_Slope, which has numerous main topics and related features that disappear. Only two features, ST_Slope = Flat and Sex = M, describe all 385 instances collected from the various datasets for predicting CVD risk. This level of detail is not sufficient for the complex data collected from various datasets. The related instances of ID 012 are too many to explain this problem. Thus, we use only 17 instances related to ID 483 to explain (Table 11).

**Table 11:** Explanations by trees and related main topics

ID	C45	DF	the {Oldpeak > 85}-tree	the {ChestPainType = ASY}-tree	the {ExerciseAngina = Y}-tree	the {ST_Slope = Flat}-tree	the {Sex = M}-tree	the {Sex = M, ChestPainType = ASY}-tree	the {Sex = M, ExerciseAngina = Y}-tree	the {Sex = M, ST_Slope = Flat}-tree
295	TP	TP					TP			
300	TP	TP	TP	TP				TP		
303	TP	TP	TP	TP				TP		
309	TP	TP		TP				TP		
313	TP	TP	FN	TP				TP		
316	TP	TP	TP				TP			
320	TP	TP	TP	TP				TP		
323	TP	TP	TP	TP						
331	TP	TP	TP				TP			
338	TP	TP	TP	TP				TP		
339	TP	TP	TP	TP				TP		
483	TP	TP	TP				TP			
606	FP	**FP**	TN	FP						
668	FP	TN								
844	FP	TN	TN							
870	FP	**TN**	TN				FP			
881	FP	FP					FP			

From Table 11, the number of related instances of ID 483 is 17. The single C4.5 tree has only one explanation for these instances. However, the descriptive forest has at least eight explanations from four main topics, two from independent and dependent trees each.

The descriptive forest can describe the instances of ID 012 in further detail and cover all features described by the single C4.5 tree. Both independent and dependent trees detail many main topics and eight related features. Therefore, using the descriptive forest is suitable for a descriptive task in a complex case. The results section of the next phase demonstrates the correctness and coverage of an explanation yielded by the descriptive forest.

The single C4.5 tree describes Instance ID 483 with four features that cover 17 instances of the CVD dataset, with false positives accounting for only five instances. However, the descriptive forest uses seven features, covering all features described by the single C4.5 tree. Moreover, the seven features are grouped into features related to the independent and dependent main topics; however, the descriptive forest still yields more complex details compared to the single C4.5 tree. The correctness and coverage of the explanation by the descriptive forest are also demonstrated in the results section of the next phase.

Instance ID 606 explains that the positive result is due to equal voting, whereas the {ChestPainType = ASY}-tree yields a superior F-measure value. Instance ID 870 explains that the negative result is due to equal voting, whereas the {Oldpeak > 85}-tree yields a superior F-measure value.

The results proving the correctness and coverage of the descriptive tasks of the descriptive forest are presented as follows.

#### Results of Section II: Comparison of correctness and coverage of explanations

In this section, we first prove the correctness and coverage of the descriptive forest for the whole CVD dataset by comparing it to those of a single C4.5 tree. Note that the accuracy, precision, and recall of instances not predicted by trees in the descriptive forest are not calculated.

Subsequently, we extend the results of the previous section by selecting all instances of the CVD dataset that match the path of a single C4.5 tree to define the classes of ID 012 and ID 483. We describe these instances using a descriptive forest. Finally, we compare the correctness and coverage of the explanations yielded by a single C4.5 tree and the descriptive forest.

The correctness and coverage of descriptive tasks can be measured by the accuracy (Correctness I), precision (Correctness II), and recall (coverage) of the entire dataset as a test dataset. The results are shown in Table 12.

From Table 12, all measures—Correctness I (accuracy), Correctness II (precision), and coverage (recall)—of the descriptive forest are superior to those obtained by a single C4.5 tree. A single C4.5 tree yields a Correctness I (accuracy) of 0.8573, a Correctness II (precision) of 0.8484, and a coverage (recall) of 0.8751. However, the quality of all descriptive-forest values is superior, yielding a Correctness I (accuracy) of 0.8747, a Correctness II (precision) of 0.8592, and a coverage (recall) of 0.9252.

**Table 12:** Comparison between a single C4.5 tree and descriptive forest for CVD-dataset accuracy, precision, and recall

Comparison	the single C4.5 tree	the {Oldpeak > 85}-tree	the {ChestPainType = ASY}-tree	the {ExerciseAngina = Y}-tree	the {ST_Slope = Flat}-tree	the {Sex = M}-tree	the {Sex = M, ChestPainType = ASY}-tree	the {Sex = M, ExerciseAngina = Y}-tree	the {Sex = M, ST_Slope = Flat}-tree	the Descriptive Forest
-True positive	459	316	367	311	377	4	330	285	339	470
-True negative	328	47	60	14	28	154	47	10	15	333
-False positive	82	44	44	41	51	2	26	29	28	77
-False negative	49	16	25	5	4	14	23	4	3	38
-Number of instances of the training dataset	918	423	496	371	460	174	426	328	385	918
-Correctness I (accuracy)	0.8573	0.8582	0.8609	0.8760	0.8804	0.9080	0.8850	0.8994	0.9195	0.8747
-Correctness II (precision)	0.8484	0.8778	0.8929	0.8835	0.8808	0.6667	0.9270	0.9076	0.9237	0.8592
-Coverage (recall)	0.9035	0.9518	0.9362	0.9842	0.9895	0.2222	0.9348	0.9862	0.9912	0.9252
-Overall quality (F-measure)	0.8751	0.9133	0.9141	0.9311	0.9320	0.3333	0.9309	0.9453	0.9563	0.8910
-Not predict	0	495	422	547	458	744	492	590	533	0

The descriptive forest comprises numerous trees trained by 19.0%–54.0% of the CVD dataset, with all trees covering 843 of 918 instances of the CVD dataset. The remaining 75 instances are defined as “no risk from the main topics discovered from this CVD dataset.” Thus, the descriptive forest can describe all 918 instances of the CVD dataset.

The {Sex = M}-tree yields high accuracy with the maximum number of “True Negative” instances. The features in the boundary nodes of this tree are {Sex = M}, {ST_Slope ≠ Flat}, {ExerciseAngina ≠ Y}, and {ChestPainType ≠ ASY}. Thus, we find that Sex = M alone is not a risk for CVDs, whereas Sex = M with co-factors ST_Slope = Flat, ExerciseAngina = Y, and ChestPainType = ASY substantially increase the CVD risk.

Moreover, we elaborate on the details presented in the previous section. The correctness and coverage of the related instances of ID 012 and ID 483 are shown in Table 13.

**Table 13:** Correctness and coverage of the related instances of ID 012 and ID 483

The comparison	Related instances of ID 012		Related instances of ID 483	
	A single C4.5 tree	The descriptive forest	A single C4.5 tree	The descriptive forest
True positive	342	340	12	12
True negative	0	11	0	3
False positive	43	32	5	2
False negative	0	2	0	0
Correctness I (accuracy)	0.8883	0.9143	0.7059	0.8824
Correctness II (precision)	0.8883	0.9164	0.7059	0.8571
Coverage (recall)	1.0000	0.9942	1.0000	1.0000

Table 13 indicates that the descriptive forest yields better correctness (I and II) than a single C4.5 tree. The coverage (recall) of the descriptive forest and a single C4.5 tree are similar, while the explanation by the descriptive forest yields significantly more detail than the single C4.5 tree.

### Results of Phase V: comparison of the descriptive quality of a descriptive forest and a single C4.5 tree for a big dataset

One characteristic of the heart disease health–indicators dataset [28] (BRFSS 2015) is that the imbalanced classes dataset only has 23,893 heart disease records (9.4%) from 253,680 records with 22 features. Thus, preprocessing steps are required before employing the WEKA program.

First, numeric features are subject to discretization to discover the association-rule tree. This dataset divides the features into four to 11 data ranges, which are excessive for rule discovery using the slope of interestingness or profitability-of-interestingness measure due to the sharply decreasing support rate. Thus, we use binary discretization, the “makeBinary” option in WEKA. The ranker search method option selects the optimal set of features in WEKA from the binary features generated from each numeric feature.

Second, to reduce the time for WEKA to construct CARs, 22 features are selected by the “AttributeSelection” filter in WEKA. One class and eight features are selected.

We found that all CARs with the class HDA = Yes yield 8,636 rules. From these rules, we discover 17 nodes of the association-rule tree, excluding the node ∅ ⇒ {HDA = Yes}. Among the 17 nodes, only one node belongs to the independent tree, the RB-7 node, and the remaining belong to dependent trees divided into six groups. These nodes are shown in Table 14.

**Table 14:** Rule nodes of the association-rule tree discovered from BRFSS 2015

Rule nodes	support	confidence
Domain Rule: ∅⇒{HDA = Yes}	0.0942	0.0942
RB1: {Age5yrs_6 = (8.5-inf)} ⇒{HDA = Yes}	0.0739	0.1533
RB2: {HighBP = Yes} ⇒{HDA = Yes}	0.0707	0.1647
RB3: {HighChol = Yes} ⇒{HDA = Yes}	0.0660	0.1557
RB4: {GenHlth_3 = (3.5-inf)} ⇒{HDA = Yes}	0.0427	0.2482
RB5: {DiffWalk = Yes} ⇒{HDA = Yes}	0.0391	0.2323
RB6: {Diabetes = high} ⇒{HDA = Yes}	0.0311	0.2229
RB7: {Stroke = Yes} ⇒{HDA = Yes}	0.0155	0.3825
RB1-1: {Age5yrs_6 = (8.5-inf), GenHlth_3 = (3.5-inf)} ⇒{HDA = Yes}	0.0319	0.3219
RB1-2: {Age5yrs_6 = (8.5-inf), Stroke = Yes} ⇒{HDA = Yes}	0.0121	0.4043
RB2-1: {HighBP = Yes, GenHlth_3 = (3.5-inf)} ⇒{HDA = Yes}	0.0345	0.3026
RB2-2: {HighBP = Yes, Stroke = Yes} ⇒{HDA = Yes}	0.0126	0.4195
RB3-1: {HighChol = Yes, GenHlth_3 = (3.5-inf)} ⇒{HDA = Yes}	0.0310	0.3075
RB3-2: {HighChol = Yes, Stroke = Yes} ⇒{HDA = Yes}	0.0112	0.4258
RB3-3: {HighChol = Yes, DiffWalk = Yes} ⇒{HDA = Yes}	0.0282	0.2877
RB4-1: {GenHlth_3 = (3.5-inf), Stroke = Yes} ⇒{HDA = Yes}	0.0097	0.4894
RB5-1: {DiffWalk = Yes, Stroke = Yes} ⇒{HDA = Yes}	0.0094	0.4713
RB6-1: {Diabetes = high, Stroke = Yes} ⇒{HDA = Yes}	0.0064	0.5006

From Table 14, all rule nodes except the domain rule are the main topics related to the class HDA = Yes, which can be used as a filter to select related records from the dataset. The 17 rule nodes yield 17 related datasets. Furthermore, the ratio of the class HDA = Yes of these datasets yields the probability of chasing problems of imbalanced datasets (Table 15).

**Table 15:** Ratio of the class HDA = Yes of datasets corresponding to rule nodes discovered from BRFSS 2015

Related dataset of rule nodes	Number of records	Number of class HDA = Yes	Ratio of class HDA = Yes
RB1	122,314	18,750	15%
RB2	108,829	17,928	16%
RB3	107,591	16,753	16%
RB4	43,651	10,835	25%
RB5	42,675	9,915	23%
RB6	35,346	7,878	22%
RB7	10,292	3,937	38%
RB1-1	25,121	8,087	32%
RB1-2	7,618	3,080	40%
RB2-1	28,917	8,749	30%
RB2-2	7,625	3,199	42%
RB3-1	25,584	7,868	31%
RB3-2	6,656	2,834	43%
RB3-3	24,884	7,160	29%
RB4-1	5,010	2,452	49%
RB5-1	5,037	2,374	47%
RB6-1	3,268	1,636	50%

Table 15 shows that the ratio of the class HDA = Yes in all related datasets to generate trees for the descriptive forest is in the 15%–50% range, whereas in the BRFSS 2015 dataset, records of the class HeartDiseaseorAttack = Yes (HDA = Yes) comprise less than 10% of the dataset. These characteristics reduce the effectiveness of the imbalanced class dataset (e.g., the accuracy of negative predictions).

The “number of records” defines the number of records related to each rule node, which form a subgroup of the dataset. These subgroups of datasets construct trees with the least PTS. The number of minNumObj and other properties of each tree are shown in Table 16.

**Table 16:** Properties of trees generated from datasets related to each rule node discovered from BRFSS 2015

The trees	minNumObj	Number of leaf nodes	Number of all nodes
Single C4.5 tree	362	7	13
RB1-tree	224	13	25
RB2-tree	362	6	11
RB3-tree	345	6	11
RB4-tree	362	6	11
RB5-tree	287	12	23
RB6-tree	611	3	5
RB7-tree	362	6	11
RB1-1-tree	224	11	21
RB1-2-tree	224	5	9
RB2-1-tree	570	10	19
RB2-2-tree	362	5	9
RB3-1-tree	246	13	25
RB3-2-tree	345	5	9
RB3-3-tree	270	9	17
RB4-1-tree	362	5	9
RB5-1-tree	106	8	15
RB6-1-tree	611	2	3

From the listings in Table 16, we can investigate the primary and related features of the trees. The single C4.5 tree has only one primary feature and five related features to describe the HDA from all 253,680 records in the dataset. In contrast, the trees of the descriptive forest give one independent primary feature and 6–16 dependent primary features, each with 1–12 related features. Taken together, these nodes describe the HDA from 253,680 records in the same dataset. This characteristic imbues the descriptive forest with high flexibility for explanation tasks. As there are many trees, only the overall quality of the descriptive task is presented in Table 17.

**Table 17:** Overall quality of the least-PTS trees and a descriptive forest generated from BRFSS 2015

Trees	Correctness I (Accuracy)	Correctness II (Precision)	Coverage (Recall)
**Single C4.5**	**0.9075**	**0.5925**	**0.0586**
RB1-tree	0.8508	0.5968	0.0815
RB2-tree	0.8393	0.5925	0.0781
RB3-tree	0.8481	0.6026	0.0728
RB4-tree	0.7618	0.5925	0.1292
RB5-tree	0.7787	0.6043	0.1374
RB6-tree	0.7859	0.6681	0.0779
RB7-tree	0.6599	0.5925	0.3556
RB1-1-tree	0.6978	0.5968	0.1891
RB1-2-tree	0.6472	0.5972	0.3909
RB2-1-tree	0.7147	0.5820	0.2028
RB2-2-tree	0.6378	0.5925	0.4376
RB3-1-tree	0.7133	0.5935	0.2145
RB3-2-tree	0.6366	0.6026	0.4301
RB3-3-tree	0.7297	0.5942	0.1908
RB4-1-tree	0.5978	0.5925	0.5710
RB5-1-tree	0.6170	0.6077	0.5286
RB6-1-tree	0.5939	0.6681	0.3753
**Descriptive Forest**	**0.9079**	**0.5930**	**0.0707**

As indicated in Table 17, the Correctness I measure (accuracy of the corresponding subgroup in the dataset) of each tree in the descriptive forest is lower than the Correctness I measure (accuracy of the whole dataset) of a single C4.5 tree. These results are caused by the imbalanced class problem. However, the Correctness II or precision results of each tree in the descriptive forest (measuring a subgroup of the dataset) are equal to or better than the Correctness II results of the C4.5 tree. These results indicate that individual trees in the descriptive forest can reduce the imbalanced class problem. Moreover, all trees working together as a descriptive forest yield superior precision scores for all measures: Correctness I (accuracy), Correctness II (precision), and coverage (recall). Thus, all trees for the last investigation (see Table 18) are generated under the default parameter minNumObj = 2. Table 18 explains why trees without the least PTS generated from related datasets, which would improve the precision and recall, are not used for the descriptive task.

**Table 18:** Single C4.5 tree vs. descriptive forest without the least PTS generated from BRFSS 2015

Topic	A single C4.5 without the least PTS	A descriptive forest without the least PTS
Number of leaf nodes	2,333	391 to 2,369
Number of all nodes	4,599	781 to 4,667
Number of True Positives	5,749	6,305
Number of True Negatives	228,728	228,769
Number of False Positives	1,059	1,018
Number of False Negatives	18,144	17,588
Correctness I (Accuracy)	0.9243	0.9267
Correctness II (Precision)	0.8444	0.8610
Coverage (Recall)	0.2406	0.2639

As shown in Table 18, the descriptive forest without the least PTS consistently outperforms the single C4.5 tree without the least PTS. Therefore, the alternative forest algorithm adequately performs classification tasks. However, the number of nodes is unsuitable for descriptive tasks. This also demonstrates that the least PTS is an excellent tool with tree techniques for descriptive tasks.

## Discussion

Our proposed method employs a decision tree technique to describe CVD risks. Many studies [4, 5, 12] have used decision tree techniques for descriptive and predictive tasks of each CVD dataset. However, our proposed method focuses on linked descriptive tasks [12] that use trees to describe hidden knowledge focusing on scientific tasks, and the ability to explain related features and class value. Stiglic et al. [12] required a clear tree structure that is not too complex to describe perspicuous knowledge from the Bioinformatics dataset. However, other studies [4, 5, 12] used one tree for each dataset, while our proposed method uses a “forest” (i.e., many trees) to describe an integrated CVD dataset.

Consistent with Leach et al. [3], we use many trees because the risks for each patient are specified by various environments, and “the greater risk for CVDs is due to disparity in risk factors.” [3] described the CVD risk in an African American women dataset, a clear dataset, with a decision tree, using one clear environment dataset to construct a single tree. Moreover, our method employs an integrated CVD dataset collected from various environment datasets and must discover many trees, that is, one tree for one clear environment dataset.

Son et al. [8] used 10-fold cross-validation to construct 10 trees. These trees were voted on to select the primary features for a prediction task, and the features were described as knowledge by the decision rules. Scheurwegs et al. [15] discovered primary features by selecting the most accurate trees through the random forest algorithm [16]. Both studies [8, 15] employed a good policy to discover primary features but employed all trees only for a prediction task, as several studies [17–19] have confirmed the efficiency of using a random forest for a prediction task. However, these trees are constructed using random features and training datasets. The trees generated from random techniques are difficult to use in a descriptive task, whereas the descriptive forest uses its combined trees for performing descriptive tasks.

Scheurwegs et al. [15] discovered the primary features of a dataset by applying the internal scoring metric to the random forest algorithm, whereas the descriptive forest discovers the primary features using an association-rule tree under constraining rules. Moreover, the ability of descriptive tasks is enhanced by applying the primary features to the combined trees.

The tree-structure generalization in [8] selects a compact tree from a rough set attribute reduced on 10-fold cross-validation. Stiglig et al. [12] generalized the tree structure by tuning the tree fitting in one screen. Moreno-Sanchez [23] generalized the tree structure by defining the maximum of tree level at level 3 of a decision tree constructed from feature-important measures. In contrast, the descriptive forest uses the least PTS to generalize the tree structure for performing descriptive tasks.

Mohan et al., Ghosh et al., and Ashri et al. [20–22] used hybrid machine learning with the random forest algorithm for classification tasks and employed a simple genetic algorithm, Relief and LASSO techniques, and an a priori algorithm, respectively, for feature selection. Our proposed techniques concern descriptive tasks using association-rule trees to determine the main topics.

Although our method still focuses on descriptive tasks, we attempt to provide a clear environment of datasets hidden in an integrated CVD dataset. Each clear dataset must be constructed using its primary features. Furthermore, we discovered hidden knowledge and primary features using an association-rule tree [25]. All trees are combined as a descriptive forest for descriptive tasks.

Moreno-Sanchez [23] used ensemble trees to describe primary features by voting and selecting the features of ensemble trees. Subsequently, these features were used to generate a new decision tree and describe the knowledge hidden in a dataset. However, we still focused on discovering many trees—each tree constructed by its primary features—from an integrated CVD dataset. To avoid arriving at a conclusion for CVD knowledge from a single tree, we extended the ability of many trees to gain explanatory knowledge of CVD risks.

Previously [24], we discovered the primary features in the dataset using an association-rule tree under a constraining rule [25]. In this previous work, these primary features are represented on a fishbone diagram. The feature extraction is based on minimum support and minimum confidence, whereas the descriptive forest uses all primary features in the trees of the forest.

In related works, various features for descriptive tasks are determined in a feature-selection step or a primary-feature discovery step. The descriptive forest provides more details for discovering both independent and dependent primary features. Owing to this characteristic, the descriptive forest achieves more flexible explanatory ability than previous methods.

A descriptive task can determine different qualitative results that are difficult to distinguish in a quantitative comparative study. As the present experimental study was based on only two datasets, it cannot clarify a quantitative comparison between the descriptive forest and a single C4.5 tree. The descriptive forest could be applied to CVDs or other disease datasets as a disease-diagnostic tool to explain the primary features and related features of new people in the “may be” style; for example, “you may be at risk of CVDs” or “you may not be at risk of CVDs.” Such primary and related features are extractable from our available database. However, many people may be undiagnosed future patients. Although the present descriptive forest is unsuitable for prediction, explaining the CVD risk will encourage these potential patients to adopt a healthy lifestyle.

The descriptive forest was evaluated only in a pilot experiment. In future works, evaluations will be extended to all stages to build the descriptive forest. We will also consider new methods for discovering primary features, a new policy for generalizing the tree structure to descriptive tasks, and a new framework for combining all trees into a descriptive forest.

However, Phase V results show that a descriptive forest without the least PTS is applicable to classification tasks. In the future, this technique may become an alternative forest algorithm for classification. Moreover, the descriptive forest can potentially reduce the imbalanced class problem.

## Conclusions

In this study, we aim to reveal many decision trees from an integrated CVD dataset. We demonstrate that the presence of many trees indicates the roles of main topics or primary features related to CVDs that disappear due to the bias of the root node of a single tree in selecting other nodes to work together. Thus, we propose a method to discover the main topics before constructing trees, where each main topic is not biased by the others, and all trees are worked together as the descriptive forest.

We apply the association-rule tree [25] to discover the main hidden topics without setting the minimum support or confidence. Thus, we discovered only one set of main topics to avoid the problem of having various sets of main topics due to setting various levels of minimum support and confidence. The main topics discovered by our proposed method comprise independent and dependent main topics, and this characteristic guides the formation of a descriptive forest. Subsequently, we used all the discovered main topics to construct trees.

All tree structures in the descriptive forest must be constructed with similar tree complexities in a consistent environment. However, the proper number of members at a leaf node that enables cooperation of all trees is difficult to define. Therefore, we used the least PTS to validate the tree-structure generalization in a consistent environment.

Consequently, all trees collectively form the descriptive forest. The descriptive forest is a suitable new tool that can flexibly explain CVD risk or risk voting. However, the trees used in the explanation cannot be overly complex, as each patient should be described by one or several trees in the descriptive forest. For explaining CVD risks, the proposed method assumes that patients come from various environments and carry different CVD-related risks.

The results showed that the descriptive forest explains the CVD risks of each patient. However, the explanations from a C4.5 tree are extremely complex and difficult to understand or accept.

We also compared the acceptabilities of the explanations derived from the descriptive forest and C4.5 tree and proved the acceptabilities in terms of the accuracy, precision, and recall of the dataset. As the comparison is meant for descriptive and not for prediction tasks, the chosen measures are not intended for predicting classes of new instances. Instead, the measures estimate the coverage and correctness of the explanations in an available dataset or the whole dataset.

Eight main topics and a descriptive forest with eight trees were discovered from 918 records of a heart failure–prediction dataset, in which 11 features were collected from five datasets [27]. This descriptive forest can explain CVD risks better than a C4.5 tree.

The comparisons between our proposed method and a single C4.5 tree show that the descriptive forest yields significantly better complex explanations than a single C4.5 tree (Phase IV, Section I).

In addition, the overall quality, correctness, and coverage of the explanations given by our proposed method are better than the overall quality of explanations given by a single C4.5 tree (Phase IV, Section II).

The strength of our proposed method lies in its ability to describe the main topics and related factors using the independent and dependent trees in a descriptive forest. Dependent trees work together as co-factors that are hard to discover. In this study, we found that Sex = M substantially increases the risk when the patient has ST_Slope = Flat, ExerciseAngina = Y, and ChestPainType = ASY as co-factors. In contrast, only Sex = M without these co-factors is not a primary feature of CVD risk.

Moreover, our proposed method works well with imbalanced classes of a large dataset, for example, 253,680 instances of the heart disease health–indicators dataset. This result demonstrates the feasibility of our method.

In summary, these results indicate that our method is suitable for descriptive tasks. This technical characteristic is important for knowledge discovery and explaining scientific data.

## Data Availability

Datasets: **Alex Teboul** 2022 Heart Disease Health Indicators Dataset. Available at https://www.kaggle.com/alexteboul/heart-disease-health-indicators-dataset This original dataset can be downloaded through signup or by registering to the Kaggle website. Under Creative Commons License version CC0,: Public Domain, a copy of this dataset can be shared with any website. For example, a copy can be downloaded directly to the github website https://github.com/doguilmak/Heart-Diseaseor-Attack-Classification/blob/main/heart_disease_health_indicators_BRFSS2015.csv (the source of this dataset is overviewed at https://github.com/doguilmak/Heart-Diseaseor-Attack-Classification). **Fedesoriano** 2021 Heart Failure Prediction Dataset. *kaggle.com*. Available at https://kaggle.com/fedesoriano/heart-failure-prediction This original dataset can be downloaded through signup or by registering to the Kaggle website and are declared as Open Database License v1.0. This license agreement intends to allow users to freely share, modify, and use the dataset while granting the same freedom to others. Thus, a copy of this dataset can be shared with the github website. For example, a copy can be downloaded directly from https://github.com/xpy-10/DataSet/blob/main/heart.csv (the source of this dataset is overviewed at https://github.com/xpy-10/DataSet/). The WEKA machine learning software: **Holmes G**, **Donkin A**, **Witten**, **IH.** 1994. Weka: a machine learning workbench. Available at https://waikato.github.io/weka-wiki/downloading_weka/ The WEKA software is declared as the GNU General Public License. Available at https://www.cs.waikato.ac.nz/ml/weka/ The WEKA logo is available under the Creative Commons Attribution-ShareAlike 2.5 License. Available at https://waikato.github.io/weka-wiki/citing_weka/

## Abbreviations

CVD(s): cardiovascular disease(s)
CARs: class association rules
BRFSS 2015: Behavioral Risk Factor Surveillance System 2015
ChestPainType: chest pain type
RestingBP: resting blood pressure
FastingBS: fasting blood sugar
resting ECG: resting electrocardiogram
MaxHR: maximum heart rate
ExerciseAngina: exercise-induced angina
ST slope: in electrocardiography, the ST slope is the slope at the ST segment connecting the QRS complex and T wave
Oldpeak: ST depression induced by exercise relative to rest
HDA = Yes: Class HeartDiseaseorAttack = Yes
HeartDiseaseorAttack: heart disease or attack
HighBP: high blood pressure
HighChol: high cholesterol
CholCheck: ever had blood cholesterol checked
BMI: body mass index
PhysActivity: physical activity
HvyAlcoholConsump: heavy alcohol consumption
AnyHealthcare: have you any health-care coverage
NoDocbcCost: could not see doctor because of cost
GenHlth: general health
MentHlth: number of days of poor mental health
PhysHlth: number of days of poor physical health
DiffWalk: difficulty walking or climbing stairs
Acc: accuracy
LN: number of leaf nodes
minNumObjs: minimum number of objects in the leaf node (parameter of the C4.5 tree constructed by WEKA)
PTS: prodigality for tree Structure. The complexity of the tree structure can be viewed as the ratio of leaf nodes to all nodes. After many experiments, we found that 2n > m is always true, where n and m are the numbers of leaf nodes and all nodes, respectively. For more complex trees, they give more prodigality for tree structures (or more values of 2n − m)
